# Automated Detection of Methane Leaks by Combining Infrared Imaging and a Gas-Faster Region-Based Convolutional Neural Network Technique

**DOI:** 10.3390/s25185714

**Published:** 2025-09-12

**Authors:** Jinhui Zuo, Zhengqiang Li, Wenbin Xu, Jinxin Zuo, Zhipeng Rong

**Affiliations:** 1 Sinopec Research Institute of Petroleum Engineering Co., Ltd., Beijing 102206, China; m17862515278@163.com; 2 Chinese Academy of Sciences, Aerospace Information Research Institute, State Environmental Protection Key Laboratory of Satellite Remote Sensing & State Key Laboratory of Remote Sensing Science, Beijing 100864, China; lizq@radi.ac.cn (Z.L.); rongzhipeng96@163.com (Z.R.); 3 Beijing Institute of Environmental Characteristics, Science and Technology on Optical Radiation Laboratory, Beijing 100854, China; xuwb@aircas.ac.cn; 4 National Engineering Research Center of Disaster Backup and Recovery, School of Cyberspace Security, Beijing University of Posts and Telecommunications, Beijing 100876, China

**Keywords:** methane leakage detection, infrared imaging, Gas R-CNN, feature pyramid network, automatic detection

## Abstract

Gas leaks threaten ecological and social safety. Non-contact infrared imaging enables large-scale, real-time measurements; however, in complex environments, weak signals from small leaks can hinder reliable detection. This study proposes a novel automated methane leak detection method based on infrared imaging and a Gas-Faster Region-based convolutional neural network (Gas R-CNN) to classify leakage amounts (≥30 mL/min). An uncooled infrared imaging system was employed to capture gas leak images containing leak volume features. We developed the Gas R-CNN model for gas leakage detection. This model introduces a multiscale feature network to improve leak feature extraction and enhancement, and it incorporates region-of-interest alignment to address the mismatch caused by double quantization. Feature extraction was enhanced by integrating ResNet50 with an efficient channel attention mechanism. Image enhancement techniques were applied to expand the dataset diversity. Leak detection capabilities were validated using the IOD-Video dataset, while the constructed gas dataset enabled the first quantitative leak assessment. The experimental results demonstrated that the model can accurately detect the leakage area and classify leakage amounts, enabling the quantitative analysis of infrared images. The proposed method achieved average precisions of 0.9599, 0.9647, and 0.9833 for leak rates of 30, 100, and 300 mL/min, respectively.

## 1. Introduction

Methane is one of the predominant non-greenhouse gases that is widely distributed in nature. The concentration of methane in the atmosphere has gradually increased at a rate of 0.5% per year over the last decade [1], making methane the largest source of radiative forcing after CO_2_ (0.97 W/m^2^) [2], and resulting in serious environmental impacts. Methane is a colorless, odorless, highly flammable gas with an explosive range of 9.5%. Poisoning or explosions caused by leaks in natural gas pipelines (whose main component is methane), petrochemical parks, and related equipment in production and life have seriously jeopardized the safety of human life and property [3]. To avoid this, leaks must be detected in time, and early emergency response programs must be implemented to reduce the hazards to a manageable level. In this area, detection of gas leaks is the key issue.

Conventional gas leak detection methods mainly focus on the manual inspection of pipelines and equipment, which not only requires a lot of manpower and materials but also takes a long time. As the number of devices, pipe distances, and complexity of the plant sites increase, work efficiency decreases. Gas leak detection methods have been developed based on specific sensors [4] (optical, electrochemical, and acoustic) to monitor process parameters. For example, the negative pressure wave method [5], acoustic method [6], and volume/mass balance method [7] assess leakage by acquiring pressure, acoustic signals, and flow rate parameters. However, most of these methods depend on the sensitivity of the sensors, proper data acquisition, and the accuracy of the mathematical model for data processing [8], and sensor-based fixed-point monitoring methods cannot meet the requirements of large-scale dynamic measurements.

With the development of infrared radiation technology, infrared optical gas imaging (OGI) has become an effective method for detecting gas leaks. The passive infrared imaging detection method does not require radiation sources or background reflections and is based on the differences in radiation between the gas and background regions. Due to the characteristics of the infrared absorption spectrum of the gas, it is represented in the infrared image as a gas plume (generally, the absorption plume is shown in black and the emission plume in white). Many researchers have studied infrared imaging for gas leak detection and have made significant progress. Li et al. [9] used a self-developed wideband infrared imaging system for gas leak detection to detect CO_2_. The image noise was reduced using an anisotropic diffusion filter, and then the frame difference method was used to mark the leakage area. Lu et al. [10] identified leaks using optical gas imaging infrared thermography in combination with an improved Gaussian mixture background model. Zheng et al. applied a four-dimensional parametric model to compensate for jitter in the OGI system and then combined cumulative integration of multiple frames with the high-order statistics (HOS) method to identify gas leakage areas [11]. Weng et al. [12] segmented the gas region in an infrared image using the frame difference method, extracted the scale-invariant feature transform (SIFT) features in the region, and used a support vector machine (SVM) for classification. Wang et al. [13] developed the first deep learning model for infrared gas detection by combining a convolutional neural network (CNN) and an OGI system for binary classification of methane leakage detection. Shi et al. [14] combined a Faster Region-based CNN (Faster R-CNN) model with the OGI technique to detect hydrocarbon leaks. Zhang et al. [15] proposed a novel method that utilizes the deep learning technique and convolutional neural networks (CNNs) to detect the leakage of VOC gas from a single-frame mid-wave infrared image. Zhou et al. [16] proposed a gas plume-constrained YOLOv11 model based on infrared imaging detection technology, named YPCN (YOLO-Plume Classification Network).

Although OGI offers a significant advantage, its detection efficiency is strongly affected by factors such as environmental conditions, operators, gas composition, leakage area, and detection distance. As a selector, the gas target cannot fully absorb the background radiation, meaning some of the background radiation enters the detection system. The absorption of background radiation by the gas is further reduced when the gas concentration or leakage is low, which, in turn, reduces the accuracy of leakage detection. Even gas compressed in a pipe only has a limited amount of gasification for cooling. In addition, the gas diffusion effect and irregular shape characteristics make it difficult to detect gas leaks in complex background environments. In 2018, Ravikumar et al. [17] analyzed the probability profiles of OGI-based CH_4_ leak detection in real-world scenarios, and the curves showed that their median and 90% detection probability limits correspond to a power-law relationship with the detection distances. However, the sensitivity of the OGI system directly determines the quality of the gas infrared image and influences the extraction and classification of gas features. Therefore, effective extraction of gas information features is the key to gas leak detection. Conventional feature extraction methods are SIFT [18], histograms of oriented gradients (HOG) [19], and so on. They need users to specify the feature extraction region of the detection target in advance, and specific targets are generally designed using fixed feature extraction templates. However, the above approach relies on strong a priori knowledge and a single task and requires tedious preprocessing operations. Efficient acquisition of gas regions and extraction of gas features in complex and changing environments are the major problems faced by conventional feature extraction methods.

With the wide application of computer vision in face recognition [20], autonomous driving [21], energy and environment prediction [22,23], and other fields, it has shown great potential. As a current mainstream machine learning algorithm, CNNs offer significant advantages in feature extraction and pattern recognition. CNNs based on weight sharing effectively simplify the model and reduce the number of weighted values, thereby reducing cumbersome preprocessing steps. To overcome these challenges, this study investigated a gas leak detection algorithm based on a convolutional neural network. Song et al. detected gas leaks in galvanized steel pipes using a CNN in combination with acoustic data by Song et al. [24]. Ning et al. [25] implemented leak detection in natural-gas pipelines by combining sensor signals with a CNN. Wang et al. [13] realized methane leak detection for the first time by combining a CNN with an infrared optical gas imaging system. With the application and extensive development of CNNs, CNN models based on object detection have emerged, such as the Region-based CNN (R-CNN) [26], Fast R-CNN [27], and Faster R-CNN [28]. Shi et al. [14] combined the conventional Faster R-CNN and OGI techniques to detect the locations of hydrocarbon leaks in infrared images, enabling the detection of gas targets. However, due to the low contrast and low signal-to-noise ratio of gas infrared images, it is necessary to develop a Gas R-CNN model for gas leak detection to improve the detection performance of gas targets, to better meet the requirements of complex environments.

To solve the above problems and improve the detection performance for gas targets, an automated methane gas leak detection method based on infrared images and Gas R-CNN was proposed in this study, to classify the leakage amount (≥30 mL/min) for the first time. Under the framework of the same task of OGI imaging, leak detection is systematically expanded from the existing “binary qualitative visual detection” to “quantitative visual detection of multi-level leakage”. First, gas leak detection experiments were conducted using an uncooled infrared imaging gas leak detection system for two scenarios and three leak rates (30, 100, and 300 mL/min) to obtain infrared gas images. Second, a Gas R-CNN gas leakage detection model was proposed for methane gas leak detection. The model proposes a multiscale feature network structure for the extraction and enhancement of leak gas features and uses region of interest (RoI) alignment to solve the problem of region mismatch due to double quantization. In the feature extraction network, feature extraction was realized by adding ResNet50 with an efficient channel attention (ECA) mechanism. The final gas features were obtained using a feature pyramid network (FPN) to fuse the semantic information from the different levels of ResNet50+ECA. Finally, the diversity of the infrared gas images was enriched using image enhancement techniques, and the data were fed into the Gas R-CNN model for gas leak detection to evaluate the effectiveness of the model.

The remainder of this paper is organized as follows. Faster R-CNN and Gas R-CNN models are presented in Section 2. The uncooled infrared imaging gas leakage detection system and the corresponding experimental setup are explained in Section 3. The detection process is explained in detail and the results are discussed in Section 4. Finally, the conclusions are presented in Section 5.

## 2. Methodology

### 2.1. Faster R-CNN

**Figure 1:**
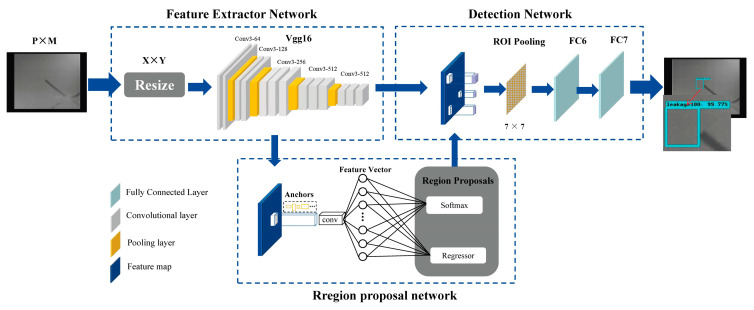
The structure of the Faster Region-based convolutional neural network (Faster R-CNN).

A faster R-CNN was developed using R-CNN [26] and Fast R-CNN [27]. It achieved the best results in the ILSVRV and COCO competitions of the year and became one of the leading target detection algorithms [28]. The main innovation of this algorithm is the use of a region proposal network (RPN) to generate RoIs instead of the selective search (SS) method, which overcomes the bottleneck in detection efficiency. Faster R-CNN consists of three modules: a feature extraction network, RPN, and detection network, as shown in Figure 1.

The feature extraction network of Faster R-CNN scales the infrared image of the gas leakage with size P × M to X × Y and then inputs the image into the CNN (typically VGG16) for feature map extraction. Next, the feature map is fed into the RPN to generate a rectangular region suggested on the feature map. RPN uses 9 anchors with width:height∈ {1:1,1:2,2:1} and size∈ {128 × 128, 256 × 256, 512 × 512} as the initial detection box for bounding box regression. The box classification layer (cls) and the box regression layer (reg) can be used to obtain information about the categories of gas objects and their corresponding coordinates in the proposal box. Here, the cls layer outputs the probability information of the object, and the reg layer outputs the parameter information (x,y,w,h) of the proposal box, including the width w, the length h, and the center coordinate (x,y). The RoI pooling layer combine feature maps and region proposals to map different-sized proposal boxes to fixed-scale feature vectors (7 × 7). Finally, the classification and regression data was fed into the fully connected layer for more accurate target detection.

### 2.2. Gas R-CNN Detection Model

**Figure 2:**
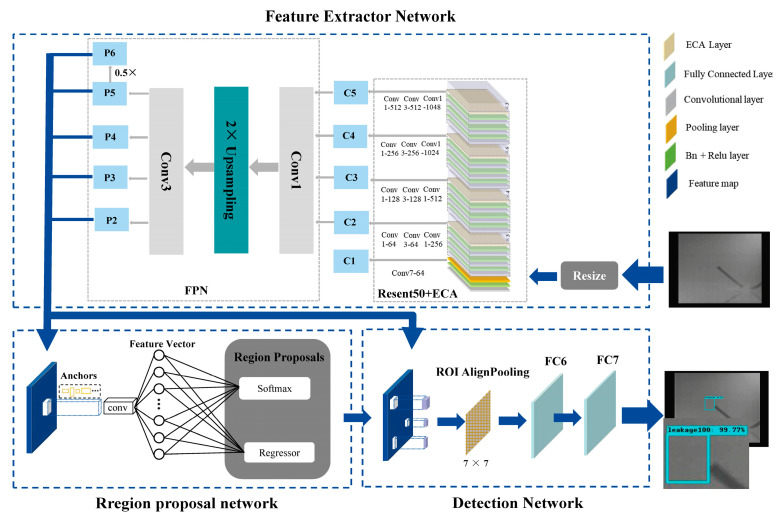
Structure of the Gas R-CNN.

In this section, an automated gas leak detection model is proposed, Gas R-CNN, as shown in Figure 2. In this study, a multiscale gas feature extraction network is used instead of VGG16 in the conventional Faster R-CNN, and RoIAlign is used instead of RoIPololong, which makes our model more skillful in detecting small gas leaks.

#### 2.2.1. Multiscale Network for Gas Feature Extraction

The observation horizon of an image can be effectively extended using convolutional networks, and different depth features of the image correspond to different levels of semantic features. Low-level features of an image provide access to rich detail and location information but also have more noise and fewer semantics. As the depth of the network increases to acquire higher-level semantic features, the feature map becomes increasingly abstract, resulting in poorer detail perception but rich semantic information [14,29,30]. Feature extraction is the key to gas leak detection. The infrared image of a gas leak is characterized by low contrast, fuzzy edges, and a low signal-to-noise ratio. At the same time, it is strongly influenced by the gas concentration and the external environment, so that the area of the gas leak is not clearly recognizable in some cases. When the gas region transitions from a shallow network to a deep network, it may be misinterpreted as a noise or background region, resulting in a loss of gas information in high-level semantics.

To solve these problems, a multiscale feature network structure for leakage gas feature extraction and enhancement was developed, as in the feature extraction network shown in Figure 2. First, feature extraction is achieved by furnishing ResNet50 with the ECA mechanism to suppress useless information; then, the FPN is utilized to fuse the semantic information. Next, the FPN is used to fuse the semantic information from different layers of ResNet50+ECA to obtain the final gas features.

(1) ResNet50 combination of ECA mechanism

To obtain better access to gas information, a residual network (ResNet) was used in the Gas R-CNN instead of VGG16. Using the ImageNet dataset, Kaiming et al. [30] demonstrated that the complexity of ResNet with a depth of up to 152 layers is still lower than that of the VGG16 network, effectively suppressing the complexity of the network while increasing its depth. The innovation of the residual block effectively solves the problems of gradient vanishing and explosions caused by deep networks. Combined with the complexity of the model and the type of gas leakage identification in this study, Resnet50 was chosen to extract the feature information, which consists of 1 fully connected layer and 49 convolutional layers.

Relevant studies have shown that the channel attention mechanism has great potential to improve the performance of DCNNs [31,32,33], but the complex attention module leads to the complexity of the model while improving the performance of the network. The ECA mechanism proposed in 2020 [34] efficiently achieves local cross-channel interactions without dimensionality reduction through one-dimensional convolution and uses a nonlinear mapping of channel dimensions to determine the size of the convolution kernel, to achieve adaptive coverage of channel interactions, which strikes a better balance between model performance and complexity. This enhances the fine-grained feature response in low-contrast, diffuse boundary scenes, avoiding the loss of detail that can come with channel compression. For low-contrast gas infrared images, the ECA mechanism helps to extract gas information, suppress useless background and noise information, and improve the performance of the Gas R-CNN.

(2) Multiscale feature fusion based on FPN

**Figure 3:**
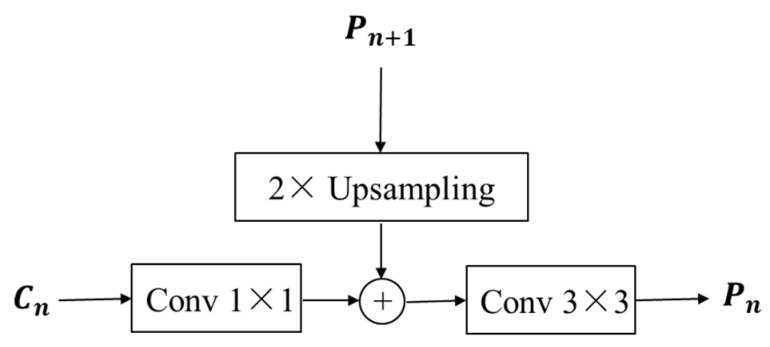
Schematic representation of the lateral connection structure.

The RPN input is the last layer of the feature map of the feature extraction network in the conventional Faster R-CNN with single feature information. The multiscale fusion approach of the FPN [35] was proposed in 2017 and proved to be accurate and fast in target detection. In the feature extraction stage, the multiscale pyramid provides a general structure to produce enhanced feature representations. With the feature pyramid networks, feature maps at each scale have strong semantic information, and the amount of calculation is greatly reduced [36]. The FPN structure consists of three main parts: bottom-up, top-down, and lateral connections. First, the bottom-up is the feature extraction process of the ResNet50+ECA network, which categorizes the feature map into five levels, from C1 to C5. Second, the top-down process upsamples the feature maps obtained at a high level and then passes them downward, passing the high-level features, which contain rich semantic information, to the low-level features. As shown in Figure 3, the lateral connection consists of three main steps: (1) The feature maps of C2–C5 reduce the dimensionality by a 1 × 1 convolution to increase the nonlinearity of the image. (2) The acquired feature Cn is fused with the upsampled acquired feature P_n+1_. (3) To eliminate the aliasing effect caused by upsampling, a 3 × 3 convolution operation was used to output the feature maps P2–P5, where P6 was obtained by downsampling P5. To improve the detection speed, the high-resolution C1 feature maps were not fed into the FPN. Therefore, the feature input of the RPN was changed from single to multiscale (P2–P6), which can effectively fuse the deep and shallow gas information and improve the accuracy of gas leak detection.

#### 2.2.2. Gas Detection Networks with RoI Align

Practical gas leak detection for tiny leaks poses a major challenge for gas leak detection. The RoI pooling layer maps the proposed region generated by the RPN to an equivalent location of the feature map to obtain the RoI. The process was acquired by selecting and offsetting the proposed regions with different sizes and proportions, but with different sizes of RoI feature maps (containing floating-point numbers). Therefore, the first quantization operation was performed to match the pixel values. Second, the fully connected layer (FC) in the detection network requires a fixed-size input that quantizes the RoI feature maps of different sizes to a fixed size (7 × 7). However, the two quantization operations cause the RoI feature map to deviate from the original image, which affects the detection of the gas leakage area and the leakage source.

**Figure 4:**
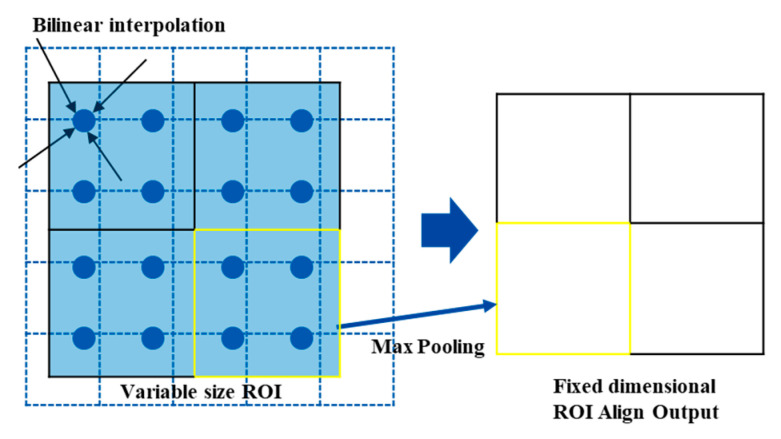
Schematic representation of RoI Align.

RoI Align Pooling [37] effectively solves the problem of mapping error and mean error caused by RoI pooling and improves the recognition accuracy of the model. As shown in Figure 4, the RoI alignment uses bilinear interpolation to calculate the exact value of multiple sampling points, and max pooling to calculate the maximum value of multiple sampling points as the final value. This replaces the quantization operation of the RoI and preserves the number of floating points. For gas leak detection tasks that require precise boundary box localization, its spatial alignment mechanism can significantly enhance localization accuracy. Therefore, RoI Align was used in this study to achieve more accurate detection and identification results.

## 3. Experimental Investigation

### 3.1. Uncooled Infrared Imaging Gas Leak Detection System

**Figure 5:**
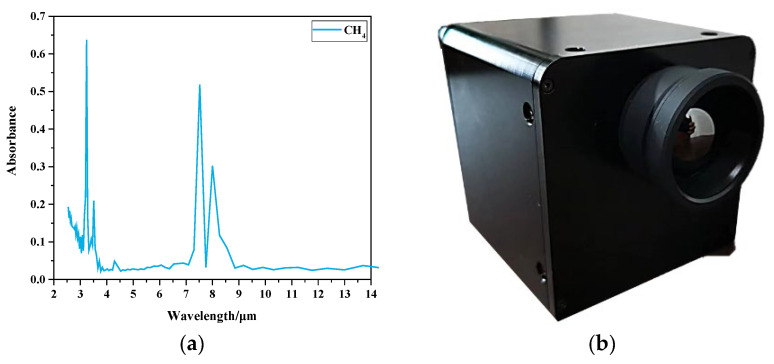
Uncooled infrared imaging gas leak detection system. (**a**) CH_4_ infrared absorption spectrum. (**b**) Uncooled infrared imaging camera for gas leak detection.

The infrared absorption spectral characteristics of the gas result in different radiation levels compared to the background, which is displayed as a moving gas cloud in the infrared image. CH_4_ is a colorless, odorless, flammable, and explosive gas. Its infrared absorption spectrum is shown in Figure 5a (infrared spectrum data from the HITRAN database). Two strong infrared absorption peaks were found, at 3.31 μm and 7.669 μm. Although the infrared absorption peak at 3.31 μm is significantly higher than that at 7.669 μm, most volatile organic compounds (VOCs) (alkanes, olefins, alkynes, etc.) have absorption peaks between 3.2 μm and 3.4 μm, and there are no significant absorption peaks for the other VOCs near 7.669 μm. In this study, the infrared spectral absorption characteristics of 7.669 μm were used for leak detection.

The in-house-developed, uncooled, infrared imaging camera for detecting gas leaks is shown in Figure 5b [38]. It uses a domestic VOx, uncooled, infrared focal plane detector (NETD: 50 mK, resolution: 640 × 480), the filter range is 7–8 μm, and the frame rate is 20 frames/s. In order to ensure that the instrument is in a stable working state for a long time, the instrument needs to be regularly maintained and calibrated in bold body. The transmission path from the background to the imaging system was divided into three layers (A, B, and C), and the radiation values of the gaseous and nongaseous paths received along the line-of-sight direction of the system are shown in Equations (1) and (2). Leakage detection was performed based on the difference between the radiation values.

### 3.2. Experimental Setup

**Figure 6:**
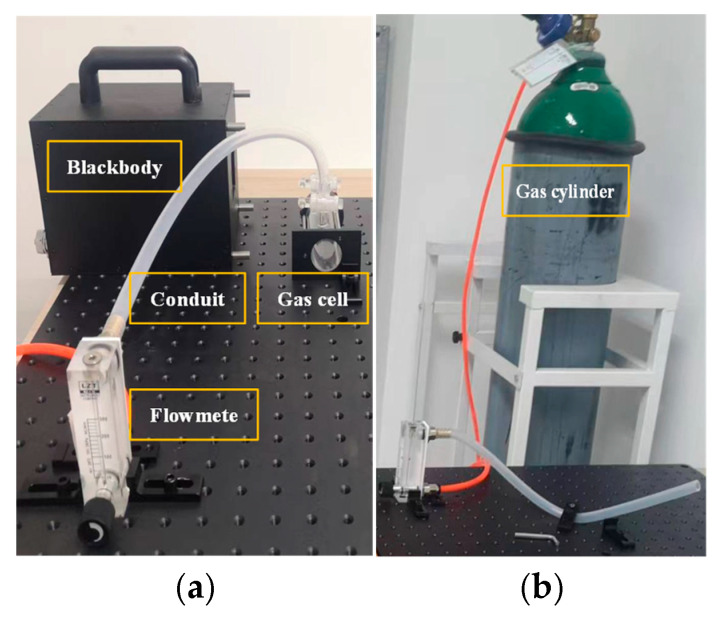
Experimental setup for indoor gas detection. (**a**) Scenario 1; (**b**) Scenario 2.

The experimental setup is illustrated in Figure 6 [39] and comprises a CH_4_ gas cylinder with a capacity of 40 L (room temperature, concentration: 1000 ppm), black body, gas cell, flowmeter, number of conduits, and fixed fittings. Image acquisition was realized using the uncooled, infrared imaging gas leak detection system described in Section 2, and the images were saved in JPG format. The software environment was Visual Studio 2015 + OpenCV 3.2.0, and the operating system was Windows 10. For this experiment, a black body and a wall were used as the background. The indoor temperature is 26 °C, and the relative humidity is 40–50%. Two leakage sources were simulated using a conduit and a gas cell, and the leakage volume was controlled with a flow meter to simulate the leakage scenarios. Scenario 1 involved a conduit source leakage with a wall in the background. Scenario 2 involved a gas cell source leak with a black body background. The experiments were performed at leak rates of 300, 100, and 30 mL/min, with a distance of 0.8 m between the system and the leak source. Each case was recorded at a frame rate of 20 fps for 4 min (repeated five times). The leaked gas may have been unstable at the beginning and end of the process. Therefore, the first 30 s and last 30 s of each video were cut out of the experiment.

### 3.3. Typical Infrared Image of a CH_4_ Gas Leak

**Figure 7:**
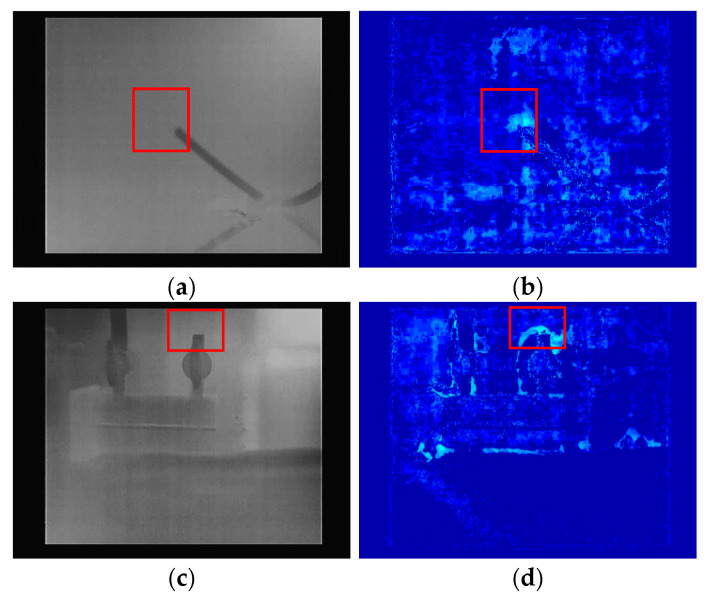
Typical infrared image of a gas leak. (**a**) Scenario 1 (100 mL/min); (**b**) Scenario 1 pseudo-color image (100 mL/min); (**c**) Scenario 2 (100 mL/min); (**d**) Scenario 2 pseudo-color image (100 mL/min).

The infrared images of the gas leakage in different scenarios of this experiment are shown in Figure 7, where the boxed parts indicate the gas leakage source and some leakage areas. The specificity of the gas target results in a low contrast in the gas infrared image, making accurate localization of the leakage source difficult under the experimental conditions. The detection of gas leaks has been realized using gas motion characteristics. However, this requires many preprocessing steps and is not able to perform large-scale detection in complex situations in a timely manner. Figure 7c,d show the pseudo-color images obtained using the motion detection method [38]. The conventional method of relying on professionals to detect the leakage source poses a major challenge in ensuring efficiency and accuracy at the same time. Therefore, a gas leak detection method is proposed based on infrared images and Gas R-CNN, which improves the efficiency and accuracy of detection.

## 4. Detection of Gas Leaks Using Infrared Images and Gas R-CNN

### 4.1. Gas Dataset

#### 4.1.1. Data Augmentation

In this section, the gas dataset is created using the infrared images of the gas leak obtained in Section 3. In this study, one image was extracted every five frames to account for the high similarity of consecutive images in the video. To better meet the practical requirements and improve the training effect of the gas leakage model, the data augmentation (DA) method was used in this study to augment the gas dataset. Each leakage image was transformed using four methods: brightness transformation, Gaussian blur, horizontal flip, and random rotation (−30–30°).

#### 4.1.2. Data Labeling

With the enhancement process described above, 9000 gas leakage datasets containing two scenarios and three leakage volumes are obtained. All images were uniformly named, and the volume and location of the leaks in each image were labeled using the LabelImg tool. The gas dataset follows the format of the PASCAL VOC2012 dataset.

#### 4.1.3. Dataset Partition

Shanmugam et al. have shown that the test time augmentation (TTA) method improves the predictive ability of the model [38]. There is a high similarity between the original image and the augmented image, which is classified into the same set to improve the training effect and generalization ability of the model. The gas dataset comprised three sets: training, validation, and test sets, divided at a ratio of 6:2:2.

### 4.2. Implementation Details

#### 4.2.1. Leak Detection

The infrared gas image was input into the Gas R-CNN model, and the processing flow was as follows. (1) The features of the gas and the background were extracted using the improved multiscale feature extraction network in the model, and the feature maps were fed into the RPN and the detection network. (2) Based on the specified intersection over union (IOU), the RPN classifies positive and negative samples and obtains region proposals and associated parameters, with positive samples indicating gas leaks (30, 100, or 300 mL/min). (3) Leaks are identified using a detection network that outputs the probability of the category while fine-tuning the region proposal box.

#### 4.2.2. Model Training

In this study, computers with specific features were used for model training, including an Intel Xeon Gold 6330 CPU @ 2.00 GHz (Intel Corporation, Santa Clara, California, USA) and a GeForce RTX 3090 GPU (NVIDIA Corporation, Santa Clara, California, USA) with 24G GB video memory and 60G GB RAM. Based on PyTorch+cuda11.3+Python3.8, the model was trained in a Linux environment using the corresponding libraries. To improve the training performance and minimize the oscillation range of the loss curve, the learning rate was set to 0.01, and the stochastic gradient descent (SGD) method was used to set the learning rate decay value to 0.0001 and the momentum factor to 0.9. As the gas did not have a fixed shape, both the confidence and IOU thresholds were set to 0.5. This is a classic setup for balancing the ratio of positive and negative samples in object detection tasks. The number of training epochs was set to 600, and the model was saved every five epochs.

#### 4.2.3. Evaluation Indicators

In this study, the performance of the gas leak detection model was evaluated using a test set. In this case, detecting a leak is a classification task, and evaluating the deviation between the predicted leak location and the original location is a regression task. Precision, recall, F1 score, average precision (AP), and mAP were used for the model performance evaluation.

Taking an image with a leak volume of 30 mL/min as an example, the recall is the proportion of samples with a leak volume of 30 mL/min that are correctly predicted. Precision is the proportion of all samples predicted to have a volume of 30 mL/min that are actually 30 mL/min. *Recall* and *precision* are defined in Equations (1) and (2),

(1) Recall=TPTP+FN

(2) $$\mathrm{Pr}ecision=\frac{TP}{TP+FP}$$

where TP denotes the number of correctly identified 30 mL/min gas leaks, *TN* denotes the number of correctly identified non-30 mL/min gas leaks, *FP* denotes the number of non-30 mL/min gas leaks identified as 30 mL/min gas leaks, and *FN* denotes the number of unidentified 30 mL/min gas leaks.

As shown in Equation (3), the *F*_1_ score harmonizes precision and recall. This can be calculated using Equation (4), when the test set contains multiple categories.

(3) F1=2×Precision×RecallPrecision+Recall

(4) $$mF_{1}={\left(\frac{1}{n}\sum_{i=1}^{n}F_{1}^{i}\right)}^{2}$$

Precision–recall (PR) curves were used to assess the differences between the performances of the different models. IOU is the degree of overlap between the manually labeled true bounding box and the model-predicted bounding box. For a specified IOU, as shown in Equation (5), *AP* is the area under the PR curve, and *mAP* denotes the average AP value of the different categories in the test set.

(5) $$AP=\int_{0}^{1}Precision(Recall)d(Recall)$$

(6) $$mAP=\frac{1}{n}\sum_{i=1}^{n}AP(i)$$

### 4.3. Results and Analysis

#### 4.3.1. Performance Evaluation of Gas R-CNN Model

**Figure 8:**
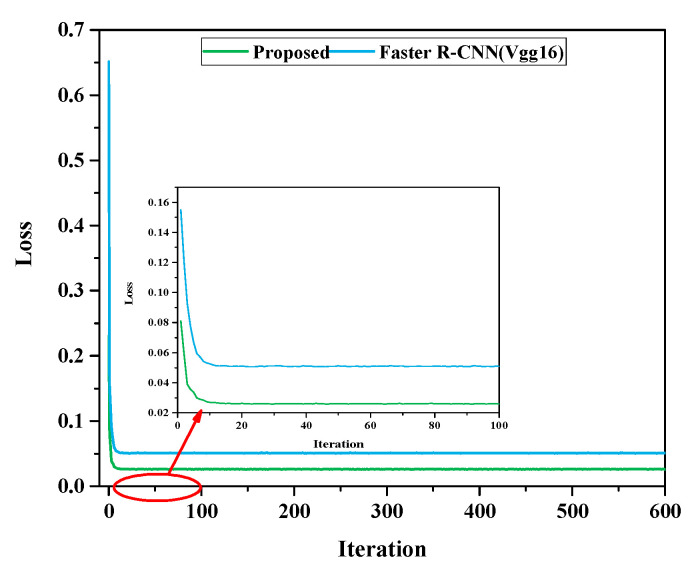
Loss curves of the conventional Faster R-CNN and the Gas R-CNN.

To evaluate the performance of the Gas R-CNN model, it was compared with the conventional Faster R-CNN model from different perspectives in this study. Figure 8 shows the loss curves of the two models after 600 iterations [40]. It can be seen that the Gas R-CNN model can be quickly reduced to 0.03 within the first 10 epochs, and the loss continues to decrease as the number of iterations increases, eventually stabilizing at 0.0262 ± 0.0001. While the conventional Faster R-CNN model drops to 0.0527 within the first 10 epochs, the value of its loss only stabilizes at approximately 0.0509 with an increasing number of iterations. The Gas R-CNN model exhibits better convergence and higher robustness.

**Figure 9:**
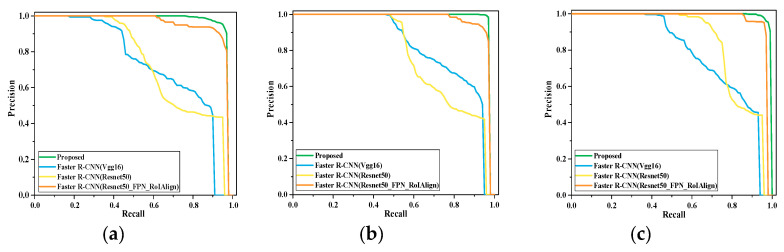
Curves for the Gas R-CNN model ablation experiments. (**a**) Leakage of 30 mL/min; (**b**) leakage of 100 mL/min; (**c**) leakage of 300 mL/min.

To evaluate the effects of Resnet50, ECA, FPN, and RoIAlign on the performance of the gas leakage detection model, ablation experiments were performed with the Gas R-CNN model. Figure 9 shows the results of comparing the PR curves of the four models. The PR curves indicate that the detection performance of each model improves under the condition of different leakage volumes, indicating the effectiveness of the improved model.

The curves of the Faster R-CNN (Resnet50_FPN_RoIAlign) model were significantly higher than those of the above two models, indicating a superior detection performance. However, the detection performance of the model decreases more clearly with a decreasing gas leakage volume. The performance of the proposed model was significantly better than those of the other three models. The detection performance remained stable with a decreasing leakage volume, further emphasizing the effectiveness of the proposed model in detecting gas leaks.

Table 1 shows the performance comparison in the ablation experiments of the Gas R-CNN model. The results show that the increase in Resnet50, ECA, FPN, and RoIAlign effectively improves the AP and mAP of gas leak detection. The AP increased by 0.234, 0.1503, and 0.2064 for leaks of 30, 100, and 300 mL/min, respectively. It can be seen that the detection of gas with low leakage is significantly improved, indicating that our model can effectively extract the gas information and also accurately identify the location of gas leakage. The F1 and mF1 of the Gas R-CNN model were also higher than those of the other models.

**Figure 10:**
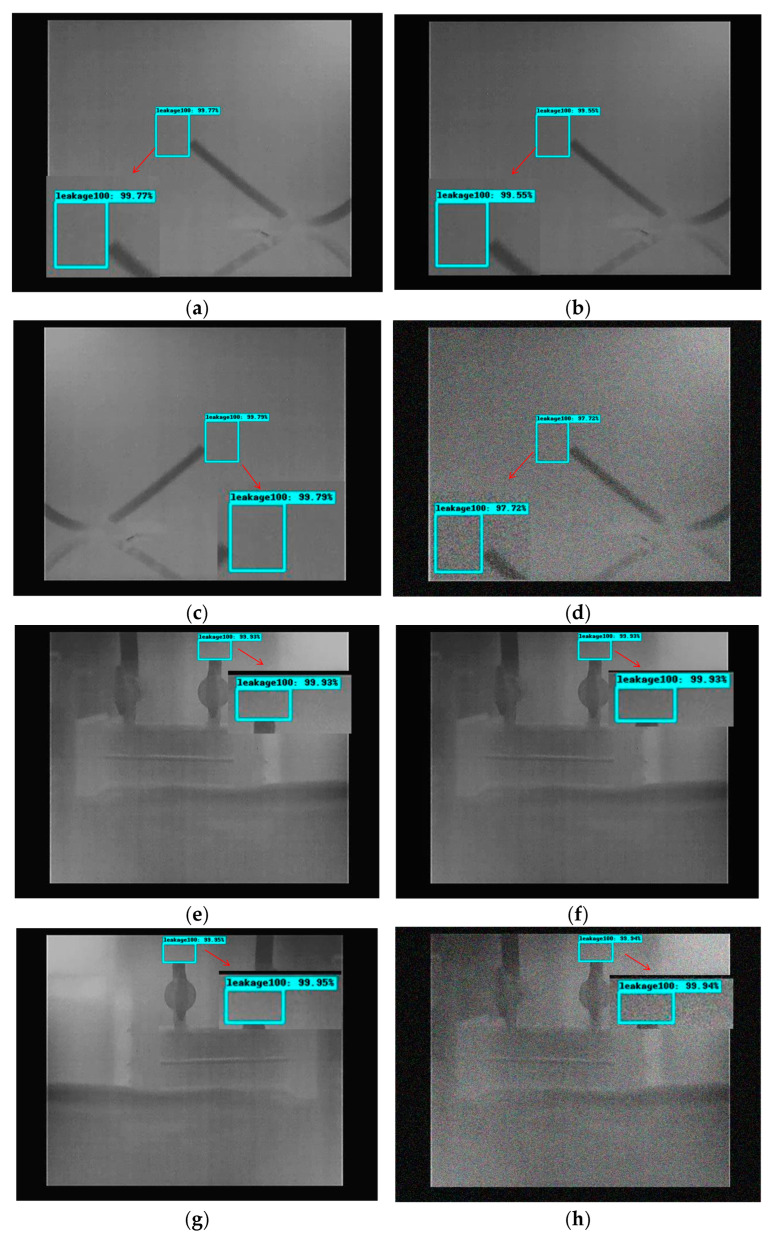
Detection results of the Gas R-CNN model: (**a**) Scene 1 (original image); (**b**) Scenario 1 (brightness transformation); (**c**) Scenario 1 (Gaussian blur); (**d**) Scenario 1 (horizontal flip); (**e**) Scenario 2 (original image); (**f**) Scenario 2 (brightness transformation); (**g**) Scenario 2 (Gaussian blur); (**h**) Scenario 2 (horizontal flip).

Figure 10 shows the detection results of the Gas R-CNN model, including the predicted leakage type and location, under four conditions for different scenarios at a leakage volume of 100 mL/min. In Scenario 1, the prediction score for the original image is 99.77%, whereas it is 97.72% for the image after Gaussian blurring. Meanwhile, in Scenario 2, the prediction score of the original image is 99.93%, whereas the prediction score of the image after Gaussian blurring it is 99.94%.

It is shown that background and environmental disturbances can lead to a degradation of the detection effect. From Table 1, it can be seen that the AP of the proposed model is 96.47% at a leakage volume of 100 mL/min, whereas the AP of the original Faster R-CNN model is 81.44%. The proposed model showed a better detection performance with high generalization ability in the overall testing environment.

#### 4.3.2. Comparison with Prevalent Models

To further evaluate the performance of the Gas RCNN model, it was compared with four typical target detection algorithms: Yolov3 [41], SSD [42], Yolovx [43], and Yolov7 [44]. The model exhibited a superior performance in different tasks and effectively extracted weak gas information, which is more satisfactory for practical applications. Quantitative analysis of the detection effect of the different models was also performed, and their AP and mAP values are listed in Table 2. Table 2 shows the superiority of the proposed model for gas detection, especially at 30 mL/min. The model effectively accounts for the fact that infrared images of gas leaks are characterized by low contrast and a low signal-to-noise ratio, with gas features often obscured by complex backgrounds. The gas information was effectively extracted, and the different leaks were classified.

#### 4.3.3. Generalization Ability of the Gas R-CNN Model

**Figure 11:**
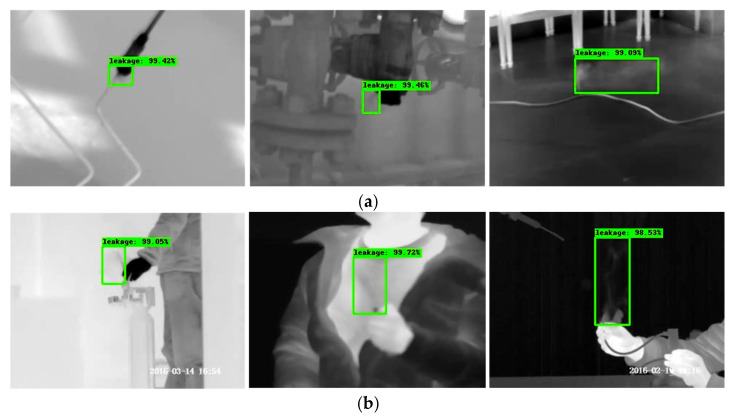
Detection results of the Gas R-CNN mode in the IOD-Video dataset. (**a**) Images of different brightness in dynamic scenarios (brighter, normal, darker). (**b**) Images of different brightness in static scenarios (brighter, normal, darker).

In this section, the IOD-Video dataset (leakage/non-leakage classification only) from the Caoxun team from Nanjing University is used to evaluate the generalization ability of the Gas R-CNN model. Figure 11 shows the detection results of the Gas R-CNN model in different environments. Due to the limitations of the IOD-Video dataset, the exact leak volumes could not be labeled. However, our model accurately identified leakage areas in different background environments. Table 3 lists the results of the quantitative analysis of the Gas R-CNN model in different environments. Six different video streams were used for testing under identical environmental conditions. The results show that the maximum AP value is 0.9920, the minimum value is 0.9591, and the mAP value is 0.9742, indicating that the method performs well in different environments. The highest recall rate was 0.9994, the lowest 0.9292, and the average recall rate 0.9735, indicating that the method can detect most leaks.

The detection of the IOD-Video dataset based on the leak/non-leak classification had a minimum AP value of 0.9591. In contrast, the detection of the gas dataset based on the leak quantity classification in this study had a minimum AP value of 0.9599. This shows that the model can maintain a reliable migration capability even when there are differences between different fields.

## 5. Conclusions

The gas detection effect is closely related to the environmental conditions, gas composition, leakage area, detection distance, and other factors. When the gas leakage is small, the gas leakage characteristics of the information are too weak to be effectively detected and identified. To solve the aforementioned problems and improve the detection performance of gas targets, an automated methane gas leak detection method was proposed based on infrared images and a Gas R-CNN, which enables gas leak classification for the first time. The Gas R-CNN model proposes a multiscale feature network structure for gas leak feature extraction and enhancement and uses region of interest (RoI) alignment to solve the problem of region mismatch due to double quantization. The experimental results show that the model successfully detects the gas leakage area and classifies the leakage amount, which enables the quantitative detection of infrared images. The APs for gas leak detection were 0.9599 at 30 mL/min, 0.9647 at 100 mL/min, 0.9833 at 300 mL/min, and 0.9693 for mAP, indicating that the proposed model has a good detection capability. The detection of the IOD-Video dataset based on the leak/non-leak classification had a minimum AP value of 0.9591.

The basic leak detection capabilities were validated in this study using the IOD-Video dataset, and we performed the first quantitative assessment of leaks using the Gas dataset, which was built in a laboratory. Within the framework of OGI imaging, leak detection is systematically extended from the existing “binary qualitative visual detection” to “quantitative visual detection across multi-level leakage.” The methodology used in this study is also applicable to other gases with infrared absorption characteristics.

However, this study has some limitations. First, more variables, such as temperature, detection distance, and gas concentration, should be included in the experiment to evaluate the effectiveness of the model more comprehensively. Secondly, there are some limitations in the laboratory conditions compared to actual leak scenarios, and the influence of external environmental factors on gas leak detection needs further investigation. In general, this study focuses on verifying methane leak detection and leakage rate classification under controllable conditions, and it does not assess the generalization in real environmental conditions. Real environments involve diverse factors, such as weather conditions (wind, rain, fog, and extreme weather) and complex backgrounds. Future work will focus on addressing these challenges.

## Figures and Tables

**Table 1:** Performance comparison in ablation experiments of Gas R-CNN.

Models	AP			mAP	F1			mF1
	30 mL/min	100 mL/min	300 mL/min		30 mL/min	100 mL/min	300 mL/min	
Faster RCNN (VGG16)	0.7259	0.8144	0.7769	0.7724	0.5511	0.6041	0.5576	0.3259
Faster RCNN (Resnet50)	0.7249	0.7628	0.8344	0.7740	0.6375	0.6905	0.6241	0.4234
Faster RCNN (Resnet50+FPN+RoIAlig)	0.9424	0.9548	0.9594	0.9522	0.9131	0.8930	0.9172	0.8241
Proposed	0.9599	0.9647	0.9833	0.9693	0.9358	0.9125	0.9755	0.8860

**Table 2:** Comparison of the results of the different detection models.

Models	AP			mAP
	30 mL/min	100 mL/min	300 mL/min	
Yolov3	0.8261	0.9417	0.9543	0.9074
SSD	0.8585	0.8693	0.8888	0.8722
Faster R-CNN (EfficientNetB7)	0.6997	0.8531	0.9179	0.8236
Yolovx	0.8758	0.9483	0.9579	0.9273
Yolov7	0.8961	0.9506	0.9694	0.9387
Proposed	0.9599	0.9647	0.9833	0.9693

**Table 3:** Gas R-CNN model detection results for different environments.

Condition	AP	SD	Recall	SD
Dynamic + Darker	0.9591	0.0410	0.9292	0.1113
Dynamic + Normal	0.9785	0.0220	0.9771	0.0476
Dynamic + Brighter	0.9609	0.0325	0.9526	0.0746
Static + Darker	0.9834	0.0031	0.9939	0.0063
Static + Normal	0.9920	0.0042	0.9994	0.0013
Static + Brighter	0.9711	0.0156	0.9887	0.0198

## Data Availability

The data generated and/or analyzed during the current study are not publicly available.

## Abbreviations

The following abbreviations are used in this manuscript:

GasR-CNN	Gas-Faster Region-based Convolutional Neural Network
ECA	Efficient Channel Attention
OGI	Optical Gas Imaging
HOS	High-Order Statistics
SVM	Support Vector Machine
CNN	Convolutional Neural Network
Faster R-CNN	Faster Region-based Convolutional Neural Network
R-CNN	Region-based Convolutional Neural Network
RoI	Region Of Interest
FPN	Feature Pyramid Network
FC	Fully Connected
VOCs	Volatile Organic Compounds
SGD	Stochastic Gradient Descent
AP	Average Precision
PR	Precision–Recall
